# Community knowledge and acceptance of indoor residual spraying for malaria prevention in Mozambique: a qualitative study

**DOI:** 10.1186/s12936-019-2653-x

**Published:** 2019-01-25

**Authors:** Amílcar Magaço, Carlos Botão, Pedroso Nhassengo, Mohomede Saide, Arminda Ubisse, Sérgio Chicumbe, Rose Zulliger

**Affiliations:** 1 Sistemas de Saúde, Instituto Nacional de Saúde, Ministério da Saúde, Moçambique, Maputo, Mozambique; 2 US President’s Malaria Initiative and Malaria Branch, Division of Parasitic Diseases and Malaria, US Centers for Disease Control and Prevention, Maputo, Mozambique

**Keywords:** Malaria, Indoor residual spraying, Acceptability, Mozambique

## Abstract

**Background:**

Malaria control remains a leading health challenge in Mozambique. Indoor residual spraying (IRS) is an effective strategy to control malaria transmission, but there are often barriers to reaching the coverage necessary for attaining maximum community protective effect of IRS. Mozambique recorded a high number of household refusals during the 2016 IRS campaign. This study sought to evaluate household and community factors related to the acceptability of IRS to inform strategies for future campaigns in Mozambique and the region.

**Methods:**

A cross-sectional, qualitative study was conducted in eight urban and rural communities in two high malaria burden provinces in Mozambique. Data were collected through in-depth interviews with community members, leaders, sprayers, and representatives of district health directorates; focus group discussions with community members who accepted and who refused IRS during the 2016 campaign; systematic field observations; and informal conversations. Data were systematically coded and analysed using NVIVO-11^®^.

**Results:**

A total of 61 interviews and 12 discussions were conducted. Community participants predominantly described IRS as safe, but many felt that it had limited efficacy. The main factors that participants mentioned as having influenced their IRS acceptance or refusal were: understanding of IRS; community leader level of support; characteristics of IRS programmatic implementation; environmental, political and historical factors. Specifically, IRS acceptance was higher when there was perceived community solidarity through IRS acceptance, desire to reduce the insect population in homes, trust in government and community satisfaction with past IRS campaign effectiveness. Participants who refused were mainly from urban districts and were more educated. The main barriers to acceptance were associated with selection and performance of spray operators, negative experiences from previous campaigns, political-partisan conflicts, difficulty in removing heavy or numerous household assets, and preference for insecticide-treated nets over IRS.

**Conclusions:**

Acceptance of IRS was influenced by diverse operational and contextual factors. As such, future IRS communications in targeted communities should emphasize the importance of high IRS coverage for promoting both familial and community health. Additionally, clear communications and engagement with community leaders during spray operator selection and spray implementation may help reduce barriers to IRS acceptance.

## Background

Malaria is one of the leading public health problems in Mozambique where the national prevalence in children between 6 and 59 months of age was 40% in 2015 [1]. Prevalence was even higher in the two most populous provinces of the country, Zambézia and Nampula, where approximately two-thirds of children under five were infected [1]. Malaria accounts for approximately 45% of outpatient clinic visits, 56% of pediatric ward admissions and 29% of all deaths in hospitals [2]. Malaria is endemic throughout the country and considered to have stable transmission, despite epidemic peaks generally related to the rainy season [3]. Important progress has been made in reducing malaria mortality in Mozambique, but the overall burden of disease reported in routine health service data has steadily increased in recent years. In 2017 there were over 9.5 million malaria cases reported throughout the country [4].

The cornerstone of malaria control in Mozambique is early diagnosis and treatment of malaria cases with effective drugs and vector control [4]. The primary malaria vector control interventions are universal distribution of insecticide-treated mosquito nets (ITNs) throughout the country and indoor residual spraying (IRS) with non-pyrethroid insecticide in districts with evidence of pyrethroid resistance, continued high transmission of malaria, and/or that are targeted for malaria elimination by regional initiatives [4]. In Mozambique, the main vectors of malaria are endophagic (feeding indoors) and endophilic (resting indoors), which makes these vectors vulnerable to ITNs and IRS [2, 4, 5]. There is now wide-scale resistance to the pyrethroid insecticides on the ITNs used in Mozambique [2, 4–7] which has underscored the importance of using more effective insecticides, whether delivered with IRS or ITNs. Presently, IRS offers a greater variety of effective insecticides for malaria control than ITNs and is a critical tool for management of insecticide resistance. As such, understanding of social factors affecting coverage and acceptance of IRS is needed.

IRS for malaria prevention has the potential to be a highly effective strategy for malaria control, particularly in the presence of pyrethroid resistance, but its effectiveness is contingent upon achieving high coverage through vectorial mass effect [8]. The World Health Organization (WHO) recommends coverage of greater than 85% of households in a community in order to have community-level protective effect [9]. IRS works to interrupt malaria transmission through killing and repelling mosquitoes, thereby reducing vector density, longevity and transmission. Importantly, it is most effective in preventing transmission of malaria after feeding, underscoring the importance of high IRS coverage at the community level in order for there to be household and individual protection. Individual and household level protection is largely derived from community level coverage [10, 11]. Thus, the WHO has emphasized the importance of community acceptance and cooperation for successful IRS implementation [9]. This is critical as a study from diverse epidemiological settings found that malaria protection from IRS was much more strongly associated with high community level coverage than with household acceptance [12].

Mozambique has a long history of IRS implementation dating back to the 1940s [13], but the NMCP has struggled in recent years to achieve the desired coverage in areas targeted for IRS in parts of the country. For example, in 2016 the reported IRS coverage of structures found by spray operators in Zambézia, the province with the highest burden of malaria in the country, ranged between 72 and 89% [14]. Given the inherent challenges in identifying the houses that should be included in the spray coverage denominator [10], the true community coverage was likely even lower. This challenge is not, however, unique to Mozambique as community level coverage was found to be poor throughout sub-Saharan Africa [11], underscoring the importance of identifying factors to facilitate better coverage.

Evidence from other African settings demonstrate that numerous factors motivate and inhibit IRS acceptance. For example, a 2010–2011 qualitative study in Tanzania found that level of education, lack of understanding of IRS effectiveness, fears of increases in other insects, potential side effects of IRS, embarrassment at removing limited household possessions, and association of the IRS campaign with politics influenced IRS acceptance [15]. A 2015 quantitative study from Uganda noted that the most important barrier to IRS acceptance was fear of adverse effects from insecticide and that age, socio-economic status, prior IRS experience, belief that IRS was beneficial and roof composition were associated with IRS acceptance [16].

Prior research in Mozambique from 2006 to 2008 in a district with a low burden of malaria found limited perceived efficacy of IRS for malaria prevention; however, participants accepted IRS in their homes based on trust and compliance with community leaders and health authorities, the perceived effect of IRS on other insects, and trusted relationships with spray operators [17, 18]. Another study in southern Mozambique of malaria prevention preferences during pregnancy noted that IRS was the least preferred prevention strategy [19].

The relevance of these data to high burden areas and in the context of next generation insecticide products is unknown. As such, this study sought to determine factors associated with IRS acceptance in the highest burden provinces of Mozambique in order to guide development of strategies to improve acceptance of IRS in future campaigns. It also sought to build upon the limited global evidence on factors that facilitate or impede IRS acceptance.

## Methods

### Study population

This qualitative study included in-depth interviews (IDI), focus group discussions (FGD), systematic field observations, and informal conversations. Data were collected in districts that were purposively selected in Nampula and Zambézia provinces based on malaria prevalence and IRS experience during the 2016 campaign. These two provinces are the most populous in the country and have the highest malaria prevalence (66% and 68%, respectively) and case burden [1]. Each district was targeted for spraying of the whole district IRS in 2016 and 2017 and had relatively high levels of refusal during the 2016 campaign. All districts used a micro-encapsulated pirimiphos-methyl insecticide in 2016 after use of the pyrethroid, deltamethrin, for the 2014 and/or 2015 campaigns. Deltamethrin was phased out in 2016 due to resistance. The IRS campaign in Zambézia province was conducted by the Ministry of Health (MoH) in partnership with the President’s Malaria Initiative while the IRS campaign in Nampula was independently implemented by the MoH. One rural and one urban study site was selected in each of the four included districts for a total of eight study sites. The two districts included in Zambézia were Mopeia (2017 population: 136,520 inhabitants) and Mocuba (422,681). The two districts included in Nampula were Nampula City (743,125) and Monapo (413,694). Data were collected in May 2017, 6 months after the end of the 2016 IRS campaign.

Participants in the study sites were purposively selected to include diverse perspectives on IRS and the IRS campaign. Participants were community members, community leaders, spray operators, and representatives of district health directorates. IDI were conducted with community members, community leaders, district representatives, and health service staff directly linked to the malaria prevention program, as they were considered to have critical and varying perspectives on IRS implementation and acceptability. FGD were conducted with individuals who refused IRS during the 2016 campaign and with individuals who accepted IRS during the 2016 campaign. A minimum of 8 individuals with the same spray status were required for a FGD in each of the sites so some study sites only had either a spray or a no spray FGD. FGD participants were different from those that participated in the IDIs.

### Data collection

This study used an ethnographic approach to data collection. This approach allowed for capturing statements and perceptions about malaria prevention and IRS, while also documenting the context in which such perspectives were expressed. In each of the study districts, the study team conducted a combination of methods, namely IDI, FGD, participant observations, and informal conversations with community leaders and members during walks around the communities to identify potential study participants. During these participant observations, team members collected field notes on behavior and comments related to the latest IRS campaign which were subsequently triangulated with IDI and FGD data.

At the start of each IDI and FGD, study researchers and research assistants administered a short, structured questionnaire to all participants to collect data on sociodemographic characteristics. This questionnaire also documented participants’ IRS acceptance or refusal. Data were collected by trained field workers using established interview guides. Interviews were conducted in Portuguese or the local language and lasted approximately 1 h. Interviews allowed participants to discuss perspectives, values and experiences in response to a series of open-ended questions. These included questions about: malaria knowledge, understanding of IRS and associated procedures, perceived importance of IRS, experience with acceptance or refusal during the 2016 campaign, the role of household heads in an IRS program, and motivators of IRS acceptance and refusal. Separate guides were used for the IDI which focused on individual perspectives and for the FGD which explored community perceptions and experiences. All interviews were recorded, transcribed and translated prior to analysis. IDIs and FGDs were conducted until the study team concluded that saturation had been reached and there was no need to interview more respondents.

### Data analysis

Data analysis was performed in two stages. Pre-analysis of the field notes led to the development of analytic categories that were analyzed sequentially during field work. This procedure helped inform the final categories that were used during the coding and analysis of full interview transcripts. NVIVO-11^®^ software was used to extract, code and sort interview text [20]. Some categories were determined prior to data collection by the interview guides, but data analysis was iterative and categories were added, removed, and amended during data analysis based on emergent study findings. The coded data were then organized into overarching themes related to the study objectives. The present analysis focuses on knowledge of IRS and motivators for IRS acceptance and refusal described by participants. Participant statements are included that are illustrative of the broader category content to contextualize study findings.

### Ethical approval

Participation in the study was voluntary and the study was ethically approved by the Institutional Committee on Bioethics in Health of the National Health Institute (012/CIBS-INS/2016). This study was reviewed by the Centers for Disease Control and Prevention (CDC) and determined to be human subjects research with non-engagement by CDC staff.

## Results

A total of 61 IDI, including community members (n = 48) and community leaders (n = 13) were conducted in the eight study sites in June–July 2017. Additionally, 12 FGD were contemporaneously conducted with 65 community members who accepted and 64 who refused IRS during the 2016 campaign.

### Participant sociodemographic characteristics

Sociodemographic characteristics of the IDI participants are summarized in Table 1. A total of 33 IDI participants refused IRS in their homes during the 2016 campaign. The median participant age was 35 years old and most respondents (79%) were self-employed in the informal market. Most of the interviewees had a primary level (n = 29) or secondary/higher level (n = 28) of education, but three respondents had no schooling. Participant characteristics were similar between those that accepted and those that refused IRS, but 92% of community leader participants accepted IRS.

**Table 1:** Characteristics of in-depth interview participants, Mozambique 2017, showing number of participants and associated percentage, except where otherwise noted

	Accepted IRS	Refused IRS	Total
N	28 (46)	33 (54)	61
*Median age (range)*	37 (19–66)	35 (19–69)	35 (19–69)
*Province*			
Nampula	17 (50)	17 (50)	34
Zambézia	11 (41)	16 (59)	27
*Community role*			
Community member	16 (33)	32 (67)	48
Community leader	12 (92)	1 (8)	13
*Sex*			
Male	16 (47)	18 (53)	34
Female	12 (44)	15 (56)	27
*Education level*			
No education	1 (33)	2 (67)	3
Primary education	15 (52)	14 (48)	29
Secondary/higher	12 (43)	16 (57)	28
College	0 (0)	1 (100)	1
*Marital status*			
Not married	9 (50)	9 (50)	18
Married	17 (50)	17 (50)	34
Cohabitating	1 (14)	6 (86)	7
Divorced/widowed	1 (50)	1 (50)	2
*Occupation*			
Formally employed	4 (36)	7 (64)	11
Informally employed	24 (50)	24 (50)	48
Unemployed/student	0 (0)	2 (100)	2

A total of 129 community members participated in twelve FGD (46 male/ 83 female). The median participant age was 34 years. The most common duration of residence in the study area was five or more years (Table 2).

**Table 2:** Characteristics of focus group discussion community member participants, Mozambique 2017

	Accepted IRS	Refused IRS	Total
N (%)	65 (50)	64 (50)	129
Median age—(range)	37 (18–66)	30 (18–60)	34
Province			
Nampula	31 (42)	42 (58)	73
Zambézia	34 (61)	22 (39)	56
Sex			
Male	27 (59)	19 (41)	46
Female	38 (46)	45 (54)	83
Duration of residence in the study area			
Native	8 (53)	7 (47)	15
< 2 years	0 (0)	2 (100)	2
3–5 years	1 (25)	3 (75)	4
> 5 years	56 (52)	52 (43)	108

### Knowledge about IRS procedures

In general, knowledge about IRS procedures and benefits among study participants was mixed. A considerable number of IDI participants reported that they understood IRS to be a method to prevent malaria and mosquito bites. Despite describing a basic understanding of IRS rationale and implementation procedures, participants frequently reported limited knowledge on how the insecticide worked, on the importance of spraying the house, and on the probable side effects or expected residual efficacy of the spray. Some respondents stated they did not trust and did not accept spraying because of the limited knowledge of these factors. Yet, higher knowledge of IRS procedures and the associated benefits was not necessarily associated with IRS acceptance as the majority of IDI participants (25 of 33) who refused IRS were able to accurately describe the procedures for spraying houses. For example, one urban man who refused IRS clearly explained the steps of IRS:

*“There you have to take your things from home. After the smell goes down you need to sweep and tidy things up again. What they put that day [the insecticide] is something that even kills, but it doesn’t kill for long.*” (IDI/Man/Refused/Urban).

Thus, it was not always a lack of knowledge of IRS procedures that led to the refusal of IRS.

One barrier to IRS knowledge mentioned by participants was that information that the IRS campaign was going to occur was often shared with community members very late, limiting their ability to prepare for the campaign. Many participants noted the need for at least a few days’ notice before the campaign in order for community members to plan and prepare for IRS compliance. This may have been more pronounced if a participant worked away from their home such as in distant agricultural fields or in formal employment.

### IRS differences by geographic area

Most IDI interviewees (n = 19) who accepted the spraying reported being happy with the results of the intervention and were glad that something was being done to combat malaria in their communities, but participants in some geographic areas felt it had limited effectiveness or safety. Participants from rural areas and participants whose homes had not previously been sprayed prior to their acceptance of the 2016 campaign were generally more satisfied and perceived IRS as more effective than those participants from urban areas. Additionally, participants from the study sites in Nampula and from the district of Mopeia in Zambézia described the 2016 campaign as efficient and improved relative to the spraying of previous years’ campaigns. For example, one participant from Mopeia reported being satisfied with the results of the last spray campaign:

*“We here in Lualua [community name] liked this year’s [IRS campaign] and are thankful for the spraying because since they have done it, so far mosquitoes are few, they are not as many as it was before. It killed not only mosquitoes, but also fleas, cockroaches and ants.”* (FGD/Woman/Accepted/Rural).

### Barriers to IRS acceptance

Despite some participants’ satisfaction with IRS, over half of participants refused IRS during the 2016 campaign. Participants who refused were mainly from urban districts with secondary and higher education level, including participants such as teachers, drivers and other public officials. These participants were skeptical of IRS and shared a desire to see a tangible impact in terms of reduced mosquitoes and malaria transmission, something they did not think occurred with IRS. The main barriers to acceptance reported by participants were associated with spray operator selection and performance, negative experiences from previous campaigns, political-partisan conflicts, difficulty in removing household assets, and preference for ITNs over IRS.

#### Spray operator selection and performance

Many participants expressed dissatisfaction with the selection of spray operators as having influenced their acceptance of the IRS campaign. According to participants, not all members of the communities that applied to work during the campaign were approved and selected for the position by the MoH and/or its implementing partners. There was little transparency as to how and why certain individuals were or were not selected. Because of this unclear process, the spraying campaign was described as having predominantly been implemented by individuals whose provenance was unknown to members of the community. Community members and leaders expressed reticence in allowing these unknown individuals into their home because they were not trusted to not damage or rob the homes.

Dissatisfaction with the selection process also led to the spread of rumours against the campaign. Community leaders noted that when young members of the community were not selected, these same individuals created rumours about IRS, encouraging residents to refuse the campaign.

*“Here the problem for some community leaders is that their children were excluded in the selection of sprayers. These children plus the leaders became upset and started to spread rumours for people not to accept”* (IDI/Community Leader/Rural).

Additionally, some study participants from the district of Mocuba in Zambézia described perceived poor implementation by IRS spray operators as one of the reasons for the limited spray effectiveness and low acceptance of spraying. Study participants alleged that spray operators diluted the insecticide used to spray the houses and then later sold the remaining insecticide. This community perception of poor spray operator performance affected trust in and acceptance of IRS implementation. In order to minimize selection of untrustworthy or unknown spray operators, participants recommended that community-based leadership be involved at all levels in the selection of sprayers to ensure better acceptability of IRS.

#### Negative experiences from previous campaigns

An important factor that influenced IRS acceptance was experience in prior campaigns. Refusal was greater among participants whose houses were sprayed in previous campaigns because they did not feel that the past campaigns were as effective as had been promised or they described collateral effects such as skin irritation and allergy in the nostrils. Additionally, many participants who refused the 2016 IRS expressed suspicions about the use of a new IRS product. Participants complained about the strong smell of insecticide and the fact that it created stains on their walls. Participants also reported experiencing frequent side effects after accepting IRS in their homes in prior campaigns such as skin allergy and irritation in the nostrils. One of the respondents who refused to spray during the 2016 campaign explained how his home was a month after spraying in a previous campaign:

*“When they do the work they dirty the walls with stains, and after 4* *weeks the mosquitoes and other insects have increased in my house, it seems that this spraying only makes things worse”* (Man/Refused/Urban).

Other participants who had homes sprayed during previous campaigns did not accept spraying in the 2016 campaign because of perceived lack of IRS effectiveness. This included the perception that IRS only chases away insects, but does not kill them. For example, a man who refused the 2016 campaign said he did not accept IRS because prior years’ IRS attracted many insects to his home. Additionally, participants noted concerns related to the residual efficacy of IRS in previous campaigns as an influence on acceptance. One participant explained, *“That product does not kill anything, mosquitoes only flee. After 3* *days or a week at the most many mosquitoes come back”* (IDI/Man/Refused/Urban).

While participants were pleased that mosquitoes and other insects declined shortly after spraying, there was some frustration with the duration of the effect of the product against mosquitoes.

*“That product helped a lot, even the cockroaches and fleas died too, but after 3* *months other mosquitoes are already coming back, it only fleas have not yet seen” (DGF/Women/Rural).*

#### Political-partisan conflicts

Another frequently reported barrier to IRS acceptance was politically-motivated rumours against IRS. These rumours were perceived to have been created by religious and community leaders not affiliated with the IRS campaign. Nampula and Zambézia are both provinces with high levels of support for the party in opposition to the current administration. In this context, many areas have both official government party community leaders and opposition party community leaders. IRS campaigns typically included government party community leaders, but not other local community leadership. The leaders who started the rumours were described as individuals with a strong influence in the community who were not involved in IRS mobilization because they were affiliated with opposition political parties.

One influential politically-oriented rumour was related to the fact that IRS was offered as a free health intervention by the ruling government party. There was a belief that the government would not offer a beneficial campaign for free in areas that did not vote for the ruling party. One participant expressed this doubt and asked, *“The government can bring this product here for free while they know that we do not vote [for their party]?”* (IDI/Man/Refused/Urban). As such, some community members believed that IRS was only provided free of charge by the government in order to hide the fact that the government was actually attempting to bring other diseases to reduce the population in groups opposed to the ruling party. These rumours were particularly common in areas where there was strong opposition party presence such as in Nampula City and in Mocuba district. Community leaders stressed their important role in either promoting or dispelling rumours related to IRS and in IRS acceptability within their communities:

*“Our problem here is more political because the population only follows what we recommend. If I say that the product is not a good thing, then many will not join”* (IDI/Community Leader/Urban).

Thus, by not including all appropriate local leadership in promoting the IRS campaign, acceptance was blocked by some community leaders, thereby affecting overall IRS acceptance. As such, community leaders emphasized the need to include community-based leadership at all levels and from various political and religious groups to dispel rumours and to facilitate higher acceptance.

#### Difficulty in removing household goods

Another barrier to IRS acceptance frequently mentioned by participants was related to the need to remove household goods for spraying. This barrier was mentioned across settings, yet important differences were noted between the rural and urban areas. In rural areas, participants frequently mentioned that they found it difficult to accept that the sprayers would see the level of poverty inside their houses when they removed their household assets for the spray. One woman explained, *“I was ashamed that they saw my poverty, if I have nothing inside it [my house]. I only have a mat and a bucket of water.”* (Female/Refused/Rural). This was in stark contrast to urban areas where participants described difficulty in removing their extensive or heavy household goods from the houses. For example, one man stated that, “*I at this age I can’t carry things from inside to the outside, so I denied”* (Man/Refused/Urban).

The level of effort associated with removing all household assets in wealthier areas was considered to be particularly cumbersome by participants who did not feel that there was a long-lasting benefit of IRS as explained by one participant: *“We cannot take our fridge and furniture outside only for something that will not last for a long time.”* (Female/Refused/Urban).

#### Preference for ITNs over IRS

Lastly, participants mentioned preference for other, non-IRS malaria prevention methods such as ITNs as one of the barriers to the acceptability of spraying. Participants from both rural and urban areas expressed reticence to accept IRS because they believed that by accepting such a method of prevention, they would not receive ITNs. One community leader explained, *“Here the people want more mosquito nets instead of spraying, so they don’t accept the spray, stating that ‘we are waiting for the nets’.”* (Community Leader/Rural). The preference for ITNs was generally because they were felt to be more effective than IRS. Another leader stated.

*“The people do not accept spraying, out of fear of not receiving nets. They say that ‘This product doesn’t work. We want mosquito nets’.”* (Community Leader/Urban).

Additionally, participants frequently reported that they perceived ITNs to be safer than IRS and, as such, preferable.

### Facilitators of IRS acceptance

The main factors influencing the acceptance of spraying were the perceived community solidarity through IRS acceptance, desire to reduce the insect population in homes, trust in government and health workers and community satisfaction with IRS effectiveness.

#### Community solidarity

The most frequently mentioned motivation for acceptance of spraying was solidarity among neighbours. The study participants reported that when a house was not sprayed, mosquitoes from that house could go to the neighbors’ homes and infect residents with malaria, underscoring the importance of IRS for community protection. Community members also influenced one-another’s acceptance by describing their positive experiences. For example, individuals who refused spraying when spray operators first came to their homes found that many of the rumours, particularly those regarding efficacy and side effects, were false after talking to neighbours. This led to acceptance when spray operators returned.

*“At first I was afraid, but after hearing the neighbours talking about killing all cockroaches, fleas and mosquitoes, then I asked for it too. When it was first sprayed, no one died, no one had side effects, so when they came to spray, no one was against it.”* (FGD/woman/accepted/urban).

The IRS campaign was accompanied by the involvement of community leaders who disseminated messages that emphasized the elimination of the mosquito that causes malaria in communities. These messages were seen as effective, but their impact, as noted above, was influenced by whether it was a trusted community leaders who delivered the messages. Participants and communities who had positive experience with spraying during previous campaigns were more likely to accept. Other interviewees whose homes were sprayed in previous rounds, but missed the most recent campaign, expressed their regret and the desire to have their home sprayed, particularly when their neighbours reported positive experiences.

#### Desire to reduce insect population of homes

Participants in the rural districts covered by the study reported having accepted IRS because of a desire to reduce the number of mosquitoes and others insects inside houses. Even though IRS was promoted by the government as a malaria prevention tool, participants were often motivated to accept because of perceived collateral benefits of IRS such as the killing of fleas.

*“I was very happy because there were lots of fleas and mosquitoes in my house. All the mosquitoes and fleas and cockroaches have already died from this spraying.”* (Woman/Accepted/Rural).

Focus group discussions, especially in rural areas, revealed that the main motivation for many participants to accept spraying was to eliminate other insects. Nevertheless, participants were also motivated by the desire to provide protection from malaria for their children by killing mosquitoes.

*“My kids used to be sick often, but now I’m happy. Since they came to do spraying they aren’t getting sick like before.”* (Man/Accepted/Rural).

#### Trust in government and health workers

Another motivation for IRS acceptance in the more remote rural study areas was the role of the government in delivering the intervention and the involvement in the campaign of influential health workers in the community. *“We accept yes, but also because it is a government order. And the government is not crazy to spend money for something that does not work.”* (Woman/Accepted/Urban). The influence of trust in the government was reinforced by another participant who stated, *“We can’t deny a thing that comes from the government, the government does not think evil, if they are for this product then it will kill mosquitoes”* (Man/Accepted/Rural).

Spray acceptance was also attributed to the fear of future problems with district health authorities in case a household member got malaria. In many cases, some of the respondents justified their decision to accept the spraying as an important perquisite for future government services.

*“We have to accept, if you don’t accept, and tomorrow go to the hospital with malaria they will send you away, they will say you are the one who denied the spraying, so you will treat yourself.”* (Woman/Accepted/Rural).

Some of these perceptions may have been propagated by spray operators and community mobilizers as some participants reported that these workers stated, *“If you do not accept it, later you cannot go to the hospital with malaria”* (Man/Accepted/Urban).

#### Community satisfaction with IRS effectiveness over time

Most participants who accepted IRS reported being satisfied with the outcome of the campaign because they had not had to worry about so many insects or seek hospital services. While some participants complained of short residual efficacy, one participant said that the effect of the IRS in his house lasted more than 6 months. Another man stated, “*For me, the mosquitoes all died, so far I can’t see anything, I can even sleep without a net, they don’t even have mosquitoes*” (Man/Accepted/Rural).

With the exception of a few heads of households in more urban areas, most of the community members covered by the study in the two provinces were willing to accept future IRS campaigns. These rural community participants stated that the last spray campaign was much better than prior years’ campaigns and was effective in combating mosquitoes and other insects.

## Discussion

IRS has been integral to many advancements in global and national malaria control, but this progress is fragile [21] and is contingent upon continued high community acceptance of IRS [11, 12]. High acceptance is critical when IRS programs use more expensive insecticides, such as the use of organophosphate in Mozambique, because as programs become more expensive they must provide high community-level coverage and benefits in order to remain cost-effective [22]. Thus, the relatively low IRS acceptance experienced by Mozambique during the 2016 campaign demonstrated the importance of understanding motivators of IRS acceptance and refusal in order to achieve public health.

This study explored community and stakeholder knowledge, experiences, and perceptions of IRS in high malaria burden districts of Mozambique. It specifically identified a number of factors that facilitated IRS acceptance or were associated with IRS refusal during the 2016 IRS campaign. The main factors that influenced IRS acceptance or refusal in this study site and others were: level of education and understanding of IRS; socioeconomic status; geographic location; community solidarity, leader influence, and preferences; IRS programmatic implementation; environmental; political and historical factors. The factors identified in this study in Mozambique and their relevance to findings from other settings are presented in Table 3.

**Table 3:** Primary facilitators and barriers to indoor residual spraying acceptance in this Mozambique study and from other settings

	Primary facilitators from this study	Primary facilitators from other settings	Primary barriers from this study	Primary barriers from other settings
Education and IRS knowledge		Community education on IRS was important influence on IRS uptake in Tanzania and Uganda [15, 16, 23] and recommended by the WHO [9]	Lack of information on spray schedule Lack of information about residual efficacy Higher education	Lack of information was a barrier in southern Mozambique [17], Tanzania [15] and Uganda [23]. In Uganda willingness was associated with higher education [16] Refusers in Tanzania were more educated [15].
Socio-economic			Families with too many/too heavy items to remove Families with too few items	Participants in Tanzania noted barrier of embarrassment of removal of limited and low quality household items [15] and in Uganda noted household property security concerns [23] Middle-class individuals in Uganda were less likely to accept IRS than individuals from lower socio-economic classes [16]
Geographic location	Rural areas		More urbanized areas	
Community	Desire to protect neighbours Learning from experience of neighbours Community leader involvement in IRS promotion	Participants in Mozambique underscored role of community in facilitating acceptance and in Tanzania noted concerns about community level effect [15, 17, 18]	Lack of community leader support Community preference for ITNs	Preference for ITNs over IRS was also noted in southern Mozambique [17–19]
Programmatic	IRS was accepted because it was believed to be effective in reducing malaria	Influence of perceived effectiveness of IRS against malaria was noted across settings [15, 16, 24] Inclusion of known spray operators was influential in southern Mozambique [18] and recommended by the WHO [9]	Selection of unknown or not trusted spray operators Concerns that insecticide was over-diluted Use of insecticide that left strong smell and stain on walls Insufficient or lack of timely communication of spray calendar	The importance of local, transparent recruitment of spray operators and their correct application of insecticide has been noted across settings [15, 18, 24–26] Skepticism regarding effectiveness of IRS persisted with some participants in Tanzania [15] Participants in Tanzania and Rwanda also noted the insecticide smell [15, 24] and in Tanzania about not having sufficient awareness of spray [15] and in Zimbabwe noted concerts with stains left on walls [27]
Environmental	Desire to remove other non-malaria insects from home	This desire was also noted in Tanzania [15], southern Mozambique [18] and Uganda [16, 23]	Belief that IRS chased away mosquitoes, but did not kill Perceived low residual efficacy	Participants in southern Mozambique, Tanzania, Rwanda and Thailand felt that IRS attracted insects and were concerned about side effects [15, 18, 19, 24, 25]
Political	Trust in government and health workers	Trust was also noted in southern Mozambique [18]	Engagement of only government party community leaders	Some participants in Tanzania felt spray was politically motivated [15]
Historical	Prior acceptance of IRS	IRS demand was higher among those with prior IRS experience in Tanzania [15] and Uganda [16]	Negative past experiences with IRS	Individuals whose houses were not sprayed in previous campaigns were less likely to accept future IRS in Uganda [16]

In Mozambique, IRS refusal was often due to barriers such as distrust in IRS spray operators, negative perceptions of IRS due to prior campaign experience or politically-motivated community rumours, lack of community leader buy-in, distrust in the government-run campaign, fear of potential side effects, and shame or difficulty associated with removing household goods. These results are similar to barriers noted in studies from other countries such as Tanzania, Uganda [15, 16, 23]. Interestingly, these findings are similar to those that were noted in lower transmission areas in Mozambique, providing some evidence that local malaria burden may not be the most important influence on IRS acceptance [9–11].

While some community members were motivated by perceived malaria-related benefits of IRS, others reported that they accepted IRS because of their desire to reduce other household insects like fleas and to comply with governmental and community expectations. Motivators of acceptance of spraying in these high transmission areas are similar to those noted for acceptance of IRS with dichlorodiphenyltrichloroethane in a low transmission area of Mozambique [9–11] which found that adherence was driven by trust in local health authorities and the influence of community leaders. This finding on the importance of community solidarity across transmission settings has implications for IRS communication strategies and overall engagement with communities. For example, key messages for future campaigns might better capitalize on this solidarity by emphasizing that by accepting IRS, community members are being good neighbours and helping to protect their communities, rather than only emphasizing individual or familial benefits. This is particularly relevant given the importance of high community acceptance for IRS to be effective. Additionally, it underscores the value of having strong buy-in from community leaders as these individuals can leverage community cohesion for the benefit of the campaign when sufficiently engaged, or can influence widespread refusal when the leaders do not have sufficient buy-in in the campaign. For example, studies from southern Mozambique found that acceptance was motivated by IRS delivery by known individuals [9–11], underscoring the important role of spray operator recruitment and selection in IRS acceptance and community leader engagement.

A notable finding of this study is that many participants did not perceive IRS to be very effective and many reported preference for ITNs, yet they continued to consent to spraying. This acceptance of IRS, despite the perception of its lack of effectiveness has also been noted in other African settings [23] and Mozambique [17, 18]. In particular, participants in this study reported concerns about the residual efficacy of IRS. This is an interesting finding given that residual efficacy of pirimiphos-methyl in the 2016 campaign in Zambézia was at least 5 months [28] and pirimiphos-methyl was considered by some participants to be more effective than the deltamethrin previously used due to the strong smell and residue left on walls temporarily after spraying.

Findings across countries underscore the importance of education and IRS knowledge on IRS acceptance. For example, participants in this study and others [15] were able to explain the IRS procedures, but they often did not understand the residual efficacy of IRS or the fact that it only killed mosquitoes that rest indoors. The low perceived effectiveness and residual effect mentioned by study participants could be related to the fact that community members often do not differentiate between the *Anopheles* mosquitoes targeted by IRS and other species of mosquito, particularly *Culex*, which tend to have greater levels of resistance to insecticides. Thus, families may not perceive the benefit of IRS if they continue to have many mosquitoes in or near their homes following the campaign, as was also noted in other studies [10]. This low perceived effectiveness underscores the complexity in communication promoting IRS acceptance. In order to promote IRS without raising false expectations, communication should specify that IRS prevents the mosquitoes that cause malaria. This nuanced message is, however, often lost since it is more complicated and less salient than messages such as the common IRS mantra in Mozambique, “*Mata o mosquito*!” (Kill mosquitoes!). Such messages may result in the short-term benefit of acceptance of the current campaign, but they may ultimately be detrimental by raising false expectations that result in negative campaign experiences that inhibit future acceptance. The important influence of historical IRS experiences on future IRS acceptance was found in this study and across settings [15, 16].

Spray operator selection and community leader buy-in were two important factors associated with IRS acceptance or refusal, a finding that may be relevant for both Mozambique and other settings. In this study, communities in which the leaders were not sufficiently engaged or where the spray operators were not known and therefore not trusted reported rumours related to IRS, perceptions that the insecticide was diluted by spray operators and fears of letting a stranger into one’s home unattended. These findings are not unique to Mozambique (e.g. [15, 18, 24–26]), and leader engagement and operators election are important areas that can be improved upon in future campaigns. The results of other studies also showed that the conduct of unknown spray operators in can be problematic and impede successful IRS campaigns or can be important motivators of acceptance. For example, the need for local, transparent recruitment of spray operators and their correct application of insecticide has been noted across settings [15, 18, 24–26]. More extensive and meaningful engagement with leaders during campaign mobilization and a more transparent spray operator selection process may enhance IRS acceptance. It is, however, important to note that in many parts of Mozambique where there are multiple community leaders from different political parties, this approach may be challenging to implement.

This study found that another barrier to IRS acceptance in poor, rural areas was a desire to not have neighbours see families lack or belongings. This finding shows that the level of poverty of some households may influence the acceptance of IRS. It is important to note, that since the majority of Mozambique’s population lives in poor, rural areas, the National Malaria Control Programme should design specific strategies for this type of population.

This study has a few important limitations that may have influenced the transferability and trustworthiness of the data. Study participants were volunteers from four high-burden malaria districts. The study findings from these areas are consistent with findings from the low-burden south of the country, but their transferability outside of Mozambique may be limited. Additionally, the study was conducted by a team of investigators from the Mozambique Ministry of Health. Many participants still mentioned concerns with the government and the government-implemented IRS campaign, but it is possible that the investigators’ background may have influenced participant responses.

## Conclusion

The findings from this study highlight important factors associated with IRS acceptance and refusal in Mozambique. Although the majority of respondents perceived the effectiveness of IRS as limited, a large proportion accepted the intervention to combat malaria due to diverse motivations such as desire to kill other insects and to comply with community and government expectations. These findings suggest that having trusted community leaders from all political parties and spray mobilizers communicate with households that IRS kills the mosquitoes that cause malaria, thereby providing protection for their families and for their communities, may increase intervention acceptance. This interpersonal communication can be challenging to implement at scale, but is necessary for ensuring household awareness and gaining community trust. Additionally, clear communication with community and political leaders during spray operator selection processes and spray implementation should help reduce barriers to IRS acceptance across diverse settings.

## Abbreviations

FGD: focus group discussion
IDI: in-depth interview
IRS: indoor residual spraying
NMCP: Mozambique National Malaria Control Programme
WHO: World Health Organization

## References

[1] MISAU, INE.Inquérito de Indicadores de Imunização, Malária e HIV/SIDA em Moçambique (IMASIDA) 2015: Relatório de Indicadores Básicos. Maputo, 2016.

[2] MISAU. Programa Nacional de Controlo da Malária: Relatório Anual, 2015. (Pública DNdS ed. Maputo, 2016.

[3] Mabunda S, Mathe G, Streat E, Nery S, Kilian A. National Malaria Indicator Survey, Mozambique (MIS-2007). Saúde Md ed. Maputo, 2007.

[4] MISAU. Programa Nacional de Controlo da Malária: Plano Estratégico da Malária 2017–2022. (Pública DNdS ed. Maputo, 2017.

[5] President’s Malaria Initiative (2017). Malaria operational plan FY 2018.

[6] Abilio AP, Marrune P, de Deus N, Mbofana F, Muianga P, Kampango A (2015). Bio-efficacy of new long-lasting insecticide-treated bed nets against *Anopheles funestus* and *Anopheles gambiae* from central and northern Mozambique. Malar J..

[7] Glunt KD, Abilio AP, Bassat Q, Bulo H, Gilbert AE, Huijben S (2015). Long-lasting insecticidal nets no longer effectively kill the highly resistant *Anopheles funestus* of southern Mozambique. Malar J..

[8] Pluess B, Tanser FC, Lengeler C, Sharp BL (2010). Indoor residual spraying for preventing malaria. Cochrane Database Syst Rev..

[9] WHO (2015). Indoor residual spraying: an operational manual for indoor residual spraying (IRS) for malaria transmission control and elimination.

[10] Bridges DJ, Pollard D, Winters AM, Winters B, Sikaala C, Renn S (2018). Accuracy and impact of spatial aids based upon satellite enumeration to improve indoor residual spraying spatial coverage. Malar J..

[11] Larsen DA, Borrill L, Patel R, Fregosi L (2017). Reported community-level indoor residual spray coverage from two-stage cluster surveys in sub-Saharan Africa. Malar J..

[12] Rehman AM, Coleman M, Schwabe C, Baltazar G, Matias A, Gomes IR (2011). How much does malaria vector control quality matter: the epidemiological impact of holed nets and inadequate indoor residual spraying. PLoS ONE.

[13] Mabaso ML, Sharp B, Lengeler C (2004). Historical review of malarial control in southern African with emphasis on the use of indoor residual house-spraying. Trop Med Int Health..

[14] PMI, Africa IRS (AIRS) Project Indoor Residual Spraying (IRS 2). Task Order Six. Mozambique: 2016 End of Spray Report. Maputo, Mozambique, Abt Associates; 2017.

[15] Kaufman MR, Rweyemamu D, Koenker H, Macha J (2012). “My children and I will no longer suffer from malaria”: a qualitative study of the acceptance and rejection of indoor residual spraying to prevent malaria in Tanzania. Malar J..

[16] Wadunde I, Mpimbaza A, Musoke D, Ssempebwa JC, Ediau M, Tuhebwe D (2018). Factors associated with willingness to take up indoor residual spraying to prevent malaria in Tororo district, Uganda: a cross-sectional study. Malar J..

[17] Montgomery CM, Munguambe K, Pool R (2010). Group-based citizenship in the acceptance of indoor residual spraying (IRS) for malaria control in Mozambique. Soc Sci Med.

[18] Munguambe K, Pool R, Montgomery C, Bavo C, Nhacolo A, Fiosse L (2011). What drives community adherence to indoor residual spraying (IRS) against malaria in Manhica district, rural Mozambique: a qualitative study. Malar J..

[19] Boene H, Gonzalez R, Vala A, Ruperez M, Velasco C, Machevo S (2014). Perceptions of malaria in pregnancy and acceptability of preventive interventions among Mozambican pregnant women: implications for effectiveness of malaria control in pregnancy. PLoS ONE.

[20] QSR International Pty Ltd. NVivo qualitative data analysis Software. In Version 11, 2016.

[21] Oxborough RM (2016). Trends in US President’s Malaria Initiative-funded indoor residual spray coverage and insecticide choice in sub-Saharan Africa (2008–2015): urgent need for affordable, long-lasting insecticides. Malar J..

[22] Chaccour C, Alonso S, Zulliger R, Wagman J, Saifodine A, Candrinho B (2018). Combination of indoor residual spraying with long-lasting insecticide-treated nets for malaria control in Zambezia, Mozambique: a cluster-randomized trial and cost-effectiveness study protocol. BMJ Glob Health..

[23] Ediau M, Babirye JN, Tumwesigye NM, Matovu JK, Machingaidze S, Okui O (2013). Community knowledge and perceptions about indoor residual spraying for malaria prevention in Soroti district, Uganda: a cross-sectional study. Malar J..

[24] Ingabire CM, Rulisa A, Van Kempen L, Muvunyi C, Koenraadt CJ, Van Vugt M (2015). Factors impeding the acceptability and use of malaria preventive measures: implications for malaria elimination in eastern Rwanda. Malar J..

[25] Hongvivatana T, Leerapan P, Smithisampan M (1982). An observational study of DDT house spraying in a rural area of Thailand. J Trop Med Hyg..

[26] Govere J, Durrheim D, la Grange K, Mabuza A, Booman M (2000). Community knowledge and perceptions about malaria and practices influencing malaria control in Mpumalanga Province, South Africa. S Afr Med J..

[27] Chimberengwa PT, Masuka N, Gombe NT, Tshimanga M, Takundwa L, Bangure D (2015). Indoor household residual spraying program performance in Matabeleland South province, Zimbabwe: 2011 to 2012; a descriptive cross-sectional study. Pan Afr Med J..

[28] PMI, Africa IRS (AIRS) Project Indoor Residual Spraying (IRS 2) Task Order Six: 2017 End of Spray Report, Maputo, Mozambique. Maputo: Abt Associates Inc.; 2018.

